# AlleleHMM: a data-driven method to identify allele specific differences in distributed functional genomic marks

**DOI:** 10.1093/nar/gkz176

**Published:** 2019-03-28

**Authors:** Shao-Pei Chou, Charles G Danko

**Affiliations:** 1 Baker Institute for Animal Health, College of Veterinary Medicine, Cornell University, Ithaca, NY 14853, USA; 2 Department of Molecular Biology and Genetics, Cornell University, Ithaca, NY 14853, USA; 3 Department of Biomedical Sciences, College of Veterinary Medicine, Cornell University, Ithaca, NY 14853, USA

## Abstract

How DNA sequence variation influences gene expression remains poorly understood. Diploid organisms have two homologous copies of their DNA sequence in the same nucleus, providing a rich source of information about how genetic variation affects a wealth of biochemical processes. However, few computational methods have been developed to discover allele specific differences in functional genomic data. Existing methods either treat each SNP independently, limiting statistical power, or combine SNPs across gene annotations, preventing the discovery of allele specific differences in unexpected genomic regions. Here we introduce AlleleHMM, a new computational method to identify blocks of neighboring SNPs that share similar allele specific differences in mark abundance. AlleleHMM uses a hidden Markov model to divide the genome into three hidden states based on allele frequencies in genomic data: a symmetric state (state S) which shows no difference between alleles, and regions with a higher signal on the maternal (state M) or paternal (state P) allele. AlleleHMM substantially outperformed naive methods using both simulated and real genomic data, particularly when input data had realistic levels of overdispersion. Using global run-on sequencing (GRO-seq) data, AlleleHMM identified thousands of allele specific blocks of transcription in both coding and non-coding genomic regions. AlleleHMM is a powerful tool for discovering allele specific regions in functional genomic datasets.

## INTRODUCTION

DNA encodes the blueprints for making an organism, in part by coordinating a complex cell-type and condition-specific gene expression program. Regulatory DNA affects gene expression by controlling the rates of a variety of steps during the transcription cycle, including opening chromatin, decorating core histones and DNA with chemical modifications, initiating RNA polymerase II (Pol II) at transcription start sites (TSSs), and releasing Pol II from a paused state into productive elongation (1). In addition, mRNAs are subjected to a host of post-transcriptional regulatory processes, most of which are influenced by the sequence of the RNA (2). How DNA or RNA sequences control each step during transcription, mRNA processing, and mRNA degradation remains poorly understood.

Finding allele specific differences in the distribution of marks along the genome is a powerful strategy for understanding the link between DNA sequence and the various biochemical processes that regulate gene expression (3,4). Diploid organisms have two copies of their DNA sequence in the same nuclear environment, providing a rich source of information about how genetic variation affects biochemical processes. Additionally, alleles in a diploid genome share the same environmental signals, cell type-specific differences within a complex tissue, and other potential confounding factors. Therefore allele specific signatures are a rigorous source of information about how DNA sequence affects gene expression.

Despite the general utility of allele specific expression measurements, surprisingly few computational methods have been proposed to detect allelic differences. Current methods that examine allele specific enrichment either test single-nucleotide polymorphisms (SNPs) independently (4,5) or combine the location of SNPs using gene annotations (6). Each of these methods has important limitations. Treating SNPs independently requires a high sequencing depth, and exhibits a bias in which regions with higher abundance of the mark of interest are much more likely to be discovered. Summing up the reads within contiguous genomic regions, such as annotated genes, can improve sensitivity and reduce bias by pooling information across SNPs that are more likely to share the same allele specificity. However, combining reads requires a well-annotated reference genome, which is not available in some species, and also prevents the analysis of marks in unannotated or non-coding regions which are critical for proper genome function.

Here, we introduce AlleleHMM, a novel computational tool that was designed to address these limitations. AlleleHMM identifies genomic blocks of SNPs that share the same allele specificity in mark abundance using a hidden Markov model (HMM). We show that AlleleHMM has significantly higher sensitivity and specificity when compared to tests that treat each SNP independently. AlleleHMM has similar statistical power compared to the practice of merging reads inside of gene annotations, and can also identify allele specific differences in unannotated non-coding RNAs when running genome-wide. When applied to publicly available global run-on sequencing (GRO-seq) data, a direct measurement of RNA polymerase, AlleleHMM discovers the location of the vast majority of genes discovered by merging gene annotations, and also identified over one thousand allele specific blocks that lie in unannotated genomic regions. Blocks of allele specific transcription are inversely correlated with the allele specific differences in repressive chromatin marks. Thus, AlleleHMM is a powerful new strategy to identify allele specific differences in functional genomic data.

## MATERIALS AND METHODS

### Overview of AlleleHMM

**Figure 1.**
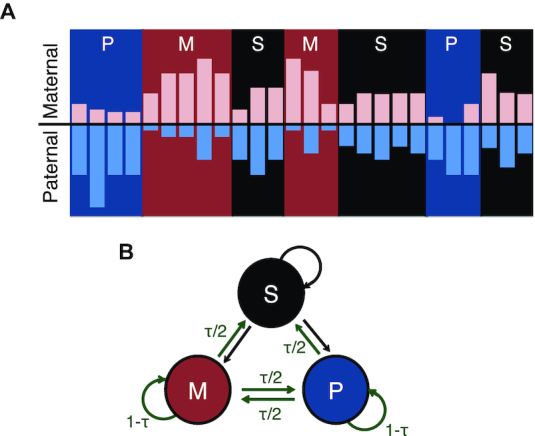
AlleleHMM uses a hidden Markov model (HMM) to infer the allele specificity of genomic markers at each SNPs. (**A**) Cartoon shows the frequency of reads mapping to the paternal (light blue bars) and maternal (pink bars) allele at positions across the genome (X-axis). Nearby SNPs show similar signatures of allele specificity depicted as blue (P, paternal allele specificity), red (M, maternal allele specificity), or black (S, no evidence of allele specificity) background identified using AlleleHMM. (**B**) The model structure of AlleleHMM. We model allele specificity using three hidden states: a symmetric state which shows no allele specificity (S, black), and states representing maternal- (M, red) or paternal-specific (P, blue) regions. SNPs can transition between hidden states. Green arrows represent the transition probabilities set using a user-adjustable tuning parameter, τ.

The primary goal of AlleleHMM is to identify allele specific blocks of signal in distributed functional genomic data. AlleleHMM relies on the key assumption that contiguous genomic regions share the same allele specificity (Figure 1A). This may happen for a variety of reasons depending on the genomic mark; for example RNA polymerase across a transcription unit shares the same allele specific differences that were derived from the rates of Pol II initiation or release from pause on the promoter which controls expression of that transcription unit (1).

We developed an HMM (7) that represents allele specificity in a distributed genomic mark using three hidden states: symmetric (S) distribution of the mark from both alleles (which shows no allele specificity), and an imbalance of the mark specific to either the maternal (M) or paternal (P) alleles (Figure 1B). AlleleHMM takes as input read counts corresponding to each allele, computed using AlleleDB (4,5). AlleleHMM uses this information to set the parameters of the HMM using Baum–Welch expectation maximization (8), save for a single holdout parameter used to tune the balance between sensitivity and specificity of AlleleHMM (see below). The Viterbi algorithm (9) is then used to identify the most likely hidden states through the data, resulting in a series of candidate blocks of signal that show evidence of allele specificity. We last calculated the coverage of allele specific read counts in each predicted AlleleHMM block and performed a binomial test to verify that the block predicted by the HMM is significantly allele specific. The last binomial test was performed to eliminate any false positives that result from multiple counts of a single read that map to multiple nearby SNPs, which are difficult to handle in the context of the HMM.

AlleleHMM can be downloaded from: https://github.com/Danko-Lab/AlleleHMM.

### HMM structure

There are three hidden states in AlleleHMM (Figure 1B): (S) a symmetric state which represents no allele specificity, and (M) or (P) which represent regions with evidence of allele specificity on the maternal or paternal allele. Each state can transition to the other two states or stay in the original state. We used allele specific read counts of SNPs with at least one mapped read as observed emissions for AlleleHMM. The distance between SNPs was not considered in the model.

### Transition probability

Transitions are permitted between all of the hidden states (Figure 1B). To control the balance between sensitivity and specificity of AlleleHMM, we used a tuning parameter, τ (Figure 1B), to limit the transition out of the M or P states. We set the transition probability from the M or P states to any of the other states to τ/2, and the transition probability to stay in the M or P state to 1-τ. The transition probability of the S state to either M or P were set using the Baum–Welch expectation maximization (EM) algorithm (7,8).

### Emission probability

Each hidden state in AlleleHMM is associated with a separate probability distribution (called the ‘emission probability’) that was used to represent the input data. The emission probabilities for all three states were calculated using the binomial distribution. The input to AlleleHMM is the total read count and maternal read count for each SNP in the genome. The input data is provided as:

\begin{equation*}\begin{array}{@{}*{6}{l}@{}} {{\rm{SNP}}:}&{1,}&{2,}&{3,}& \ldots &{{{\rm{l}}_{\rm{c}}}}\\ {{\rm{Total\,read\,count}}:}&{{{\rm{n}}_1},}&{{{\rm{n}}_2},}&{{{\rm{n}}_3},}& \ldots &{{{\rm{n}}_{\rm{l}}}}\\ {{\rm{Maternal\,read\,count}}:}&{{{\rm{x}}_1},}&{{{\rm{x}}_2},}&{{{\rm{x}}_3},}& \ldots &{{{\rm{x}}_{\rm{l}}}} \end{array}\end{equation*}

Given these input data, the emission probability for state *j* is defined as:

\begin{eqnarray*}{\rm{\, }} && \prod\nolimits_{{\rm{autosomes}}} \, \prod\nolimits_{i\ = \ 1}^{{l_c}} {\frac{{{n_i}!}}{{{x_i}!\left( {{n_i} - {x_i}} \right)!}}}\nonumber p_j^{{x_i}}{{\left( {1 - {p_j}} \right)}^{\left( {{n_i} - {x_i}} \right)}} \end{eqnarray*}

The value ${p_j}$ for each state (S, M, and P) was estimated from the data using the EM algorithm, as described below.

### Learning procedure for transition and emission probabilities

We use the Baum–Welch EM algorithm (8) to learn the transition and emission parameters in AlleleHMM (with the exception of the user adjustable tuning parameter, τ). The EM algorithm uses the forward-backward algorithm to compute the probability of each state at every heterozygous SNP in the genome with one or more mapped read (E-step). These probabilities are used to maximize the likelihood of the model given the input data (M-step). After learning model parameters using EM, we identify the most likely path of hidden states at every mapped heterozygous SNP using the Viterbi algorithm (9). These algorithms are described in detail by Durbin *et al.* (7).

### Tuning parameter optimization using GRO-seq data

AlleleHMM defines a user-adjustable tuning parameter, τ, that controls the balance between sensitivity and specificity by limiting the frequency of transitions between model states. Intuitively, users can think about τ as the threshold *P*-value that must be overcome by the input data to support a state transition.

The optimal value of τ depends on the type of data, the sequencing depth, and the heterozygosity of the genome of interest. To determine the optimal value of τ for GRO-seq data in this manuscript, we assumed that changes in allele specificity should usually arise near a transcription start site (TSSs) (Supplementary Figure S1A), because allele specificity across a gene is controlled by the rate of initiation and release from pause at the TSS (1). We evaluated the proportion of AlleleHMM blocks that start within a fixed distance of a TSS defined using dREG (discriminative regulatory-element detection from GRO-seq) (10,11) over a range of τ in the dataset of interest. As τ increased, a larger fraction of AlleleHMM blocks occurs within a predefined distance of a dREG annotated TSS (Supplementary Figure S1B). We selected the τ for each dataset at which this value approached a saturation point. For instance, in the 129/*castaneus* F1 hybrid mouse embryonic stem cells (mESC) dataset, as τ approached 1e-05, the fraction of AlleleHMM blocks beginning within 5 kb of a dREG site saturated at ∼50% (Supplementary Figure S1B, black line, Supplementary Figure S1C). Although the value of saturation varied with different window sizes, the value of τ that defined the saturation point was fairly well conserved over a reasonable range of τ (Supplementary Figure S1B). In analyses that follow we fixed τ to 1e-05 (full GM12878 dataset, mESC dataset). For subsampled GM12878, the same criteria used above suggested an optimal value of τ as 1e-04 (50% of total reads), 1e-03 (25% of total reads), and 1e-2 (12.5 and 6.25% of total reads).

To provide an orthogonal validation for the value of τ selected using the TSS strategy described above, we measured the sensitivity and specificity of AlleleHMM over different values τ in each dataset, using gene annotations as a gold-standard (see below; Supplementary Figure S1D–F). We found that setting τ using the saturation point of TSSs generally produced a sensitive model with a specificity near 1.0, as desired for the problem of discovering allele specific blocks in the unbalanced setting of an entire genome.

### Preparing data for input to AlleleHMM

Allele specific read counts were computed using AlleleDB (4,5) with some modifications. Briefly, reads were mapped to paternal and maternal genomes separately using Bowtie (14). Reads with ambiguous mapping bias were removed, following the procedure in AlleleDB (5). The Bowtie output of each parental genome was merged and each read was assigned to either the paternal or maternal haplotype based on the amount of difference between the read and each individual genome. Reads that differed from both individual genomes by the same amount were assigned randomly to one of the individual genomes. The merged Bowtie output was separated to plus strand and minus strand using in house scripts because AlleleDB was designed for non-strand-specific datasets. The allele specific read counts of each SNP on each strand were computed using AlleleDB (4,5). The AlleleDB output was further parsed into a tab-delimited table as shown in Supplementary Table S1.

An example input file can be found here: https://github.com/Danko-Lab/AlleleHMM/blob/master/input_file_exmaples/AlleleHMM_input.txt

### Identify allele specific transcribed blocks

We used the Viterbi algorithm (9) to identify the most likely set of states (M, P or S) at each heterozygous position in the genome of interest. Nearby SNPs sharing the same hidden state were stitched into blocks. We then calculated the coverage of reads in each block as follows: Reads were mapped to the diploid genome using Bowtie (12) as implemented in AlleleDB (5). The Bowtie output, including reads and their mapping position, were separated into maternal- and paternal-specific text files. Then, the coordinates were transferred to the appropriate reference genome (mm10 or hg19) using liftOver (13). We used the older hg19 human reference genome because existing fully phased personal references for each of the two GM12878 haplotypes were available in this coordinate system (4,5), but not in the newer hg38. We calculated the number of reads from the maternal or paternal haplotype in each AlleleHMM block using bedtools (14). Binomial tests were performed for each block and the false discovery rate was estimated to correct for multiple hypothesis testing (15). These steps were performed to eliminate false positives derived from multiple counts of a single read that mapped to multiple nearby SNPs, which were more complicated to consider in the context of our HMM. AlleleHMM outputs two bed files: one with all blocks and the other only reports the blocks with an FDR ≤10% as significantly allele specific.

### Performance test with synthetic data

**Figure 2.**
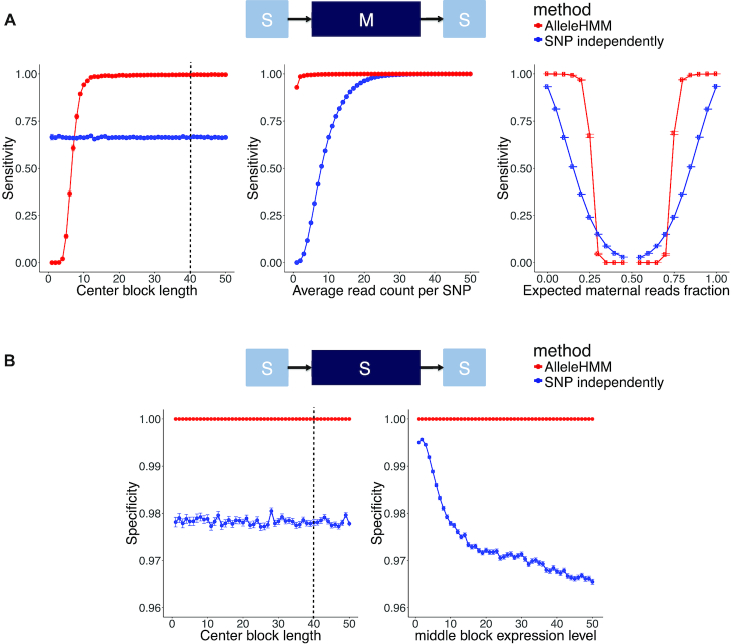
AlleleHMM had better sensitivity and specificity compared with standard methods performing independent binomial tests for each SNP. (**A**) Scatterplots show the sensitivity for each SNP in the center block of AlleleHMM (red) and independent binomial tests (blue) as a function of the length of a maternal specific blocks (the number of continuous SNPs sharing same allele specificity, left), the average read count at each SNP (center), or the expected maternal reads fraction (right). Error bars represent the standard error of 1000 independent simulations. The dotted line indicates the average number of SNPs per human gene. (**B**) Scatterplots show the specificity of AlleleHMM (red) and independent binomial tests (blue) as a function of the length of the symmetric middle block (the number of continuous SNPs sharing same allele specificity, left) or the average read count at each SNP (right). Error bars represent the standard error of 1000 independent simulations. The dotted line indicates the average number of SNPs per human gene.

To test how the performance of AlleleHMM compared with current standards in the field, which involve testing SNPs independently, we developed a simulation strategy. We used a simulation strategy because the location and magnitude of allele specific blocks could be controlled precisely, providing a confident ground truth dataset for rigorous performance evaluation. The synthetic data was composed of three blocks, each representing a region with allele specificity as shown on the top of Figure 2. The flanking blocks were symmetric, and were simulated using parameters that were kept consistent throughout the study.

The following parameters were changed to simulate the middle block with allele specific transcription: length, expression level, and the degree of allele specificity. Length was defined as the number of contiguous SNPs sharing the same allele specificity, and was set to 100 when testing other parameters, settings that were within the range of a typical gene in F1 hybrid mice (Supplementary Figure S2A, top). Expression level, or the average read count per SNP in the block, was set to 10 when testing other parameters. The degree of allele specificity was defined as the probability that a read comes from the maternal allele in a binomial or beta-binomial event, and was set to 0.9 when testing other parameters. The total read counts of each location were simulated with a Poisson distribution and the allele specific read count was simulated by either the binomial or beta-binomial distribution with overdispersion of 0.25. The overdispersion of 0.25 was chosen based on the estimates of two real data sets: GRO-seq in GM12878 and 129/*castaneus* F1 hybrid mESCs. The estimate was performed in R using the VGAM library (16) using all SNPs covered by at least 5 reads. The parameters used are summarized in Supplementary Table S2.

### Performance test with real biological data

To test the performance of AlleleHMM with GRO-seq data, we applied AlleleHMM and independent binomial tests implemented in AlleleDB to GRO-seq from GM12878 and 129/*castaneus* F1 hybrid mESCs. The allele specificity of each GENCODE gene annotation was estimated and used as a surrogate for the ground truth. Allele specific reads within each GENCODE gene annotation were counted using bedtools (14). Binomial tests were then performed for each gene annotation and the false discovery rate was used to correct for multiple hypothesis testing. We used Release 28 (mapped to hg19/ GRCh37) for GM12878 and Release M17 (mm10/ GRCm38.p6) for 129/*castaneus* F1 hybrid mouse. The sensitivity, specificity, and precision were calculated at the SNP-level. All SNPs inside GENCODE gene annotations with at least one read mapped were used. The allele specificity was determined by AlleleHMM, independent binomial tests, and using GENCODE gene annotations were summarized and used to calculate performance using in-house scripts.

### Comparison with H3K27me3 ChIP-seq data

To test the correlation between transcription and H3K27me3 in GM12878, we mapped the H3K27me3 ChIP-seq reads to the diploid genome of GM12878 using Bowtie as implemented in AlleleDB (5). The Bowtie output, including reads and their mapping positions, was separated into maternal- and paternal-specific files. Coordinates were transferred to the reference genome (hg19) using liftOver. We used bedtools coverage to calculate the number of H3K27me3 ChIP-seq reads falling into each AlleleHMM block obtained from GRO-seq in GM12878. We then calculated the ratio of maternal-specific and paternal-specific H3K27me3 ChIP-seq reads in each GRO-seq AlleleHMM block and summarized using in-house R scripts.

### Data used in this study

GRO-seq of 129/*castaneus* F1 hybrid mouse embryonic stem cells: SRA ID number SRR4041366.
GRO-seq of GM12878: SRA ID number SRR1552485
H3K27me3 ChIP-seq data of GM12878 were fastq files from ENCODE: ENCFF000ASV, ENCFF000ASW, ENCFF000ASZ, ENCFF001EXM, ENCFF001EXO

### Software availability

The source code of AlleleHMM is available under the BSD 2-clause license https://github.com/Danko-Lab/AlleleHMM
Scripts used for each computation are available at: https://github.com/Danko-Lab/AlleleHMM/tree/master/analysis_for_AlleleHMM_manuscript

## RESULTS

### Finding allele specific differences using a hidden Markov model

Differences in mark abundance between two heterozygous alleles are often correlated across multiple adjacent SNPs (Figure 1A). We developed AlleleHMM to identify genomic regions that share allele specific differences in functional mark abundance. AlleleHMM takes as input counts of reads mapping unambiguously to each of the two alleles in heterozygous positions of a phased reference genome. AlleleHMM models the data using a hidden Markov model (HMM) that divides the genome among three hidden states: a symmetric state (state S) which shows no allelic difference in mark abundance, and regions with a higher signal on the maternal (M) or paternal (P) allele (Figure 1B; see Materials and Methods). AlleleHMM models the distribution of read counts mapping to each allele using a binomial distribution. To control the tradeoff between sensitivity and specificity, we introduced a user-adjustable tuning parameter, τ, that constrains the transition probability out of either the maternal or paternal state. Aside from τ, all other model parameters are set using expectation maximization over the provided data.

### Performance test with binomial-distributed simulated data

To determine how AlleleHMM performed in practice, we simulated blocks of contiguous SNPs with specific levels of allele specificity. We simulated a sequence of SNPs composed of three blocks using the binomial distribution (Figure 2A, top): two blocks with equal signal in both alleles and one middle block that exhibited a known difference in signal between alleles. We evaluated the performance of AlleleHMM after we systematically changed the length, signal level, and degree of allele specificity in the middle block holding other parameters constant (see methods) (Figure 2A). We evaluated the accuracy of AlleleHMM by examining the sensitivity (the fraction of true positives recovered). We also examined the specificity (the fraction of correctly classified true negatives) and precision (the fraction of true positives over all positive calls) of AlleleHMM, incorporating simulations in which the middle block was symmetric to compute precision. We chose simulation parameters characteristic of global run-on and sequencing (GRO-seq) (17), a direct measurement of RNA polymerase which is challenging to use in allele specific measurements because only a few reads map to each SNP in a typical dataset, resulting in poor statistical power.

AlleleHMM identified allelic differences in simulated data with higher sensitivity, specificity, and precision compared with simple methods that perform independent binomial tests at each SNP (Figure 2; Supplementary Figure S2A). The sensitivity of AlleleHMM for each simulated allele specific SNP in the center block increased with block length. AlleleHMM had a higher sensitivity than independent binomial tests when the center block contained as few as 8 adjacent SNPs, and a higher precision with only 10 adjacent SNPs (Figure 2A, Supplementary Figure S2A, left), shorter than observed in most mammalian genes (on average, 39.7 SNPs per gene for human CEPH Utah and 237.2 SNPs for 129/*castaneus* F1 hybrid mouse, Supplementary Figure S2B). AlleleHMM had a higher sensitivity across the spectrum of signal levels (Figure 2A, center). Likewise, we found that AlleleHMM was more sensitive across an important range of allele specificity magnitudes (≤0.25 or ≥0.75; Figure 2A, right). Treating SNPs independently resulted in a higher sensitivity when the allele specificity was much lower in magnitude (0.25–0.75), which we attribute to the presence of rare individual SNPs that have a higher magnitude of allele specificity than the average for that block due to random statistical fluctuations. AlleleHMM had a higher specificity throughout the range of expression and block length parameters than treating SNPs independently (Figure 2B), and generally had superior precision as well (Supplementary Figure S2A), demonstrating that AlleleHMM does not trade a higher sensitivity for a lower specificity. Thus we conclude that AlleleHMM had better sensitivity and specificity for allele specific transcription in synthetic data simulated using the binomial distribution.

### Performance test with overdispersed synthetic data

**Figure 3.**
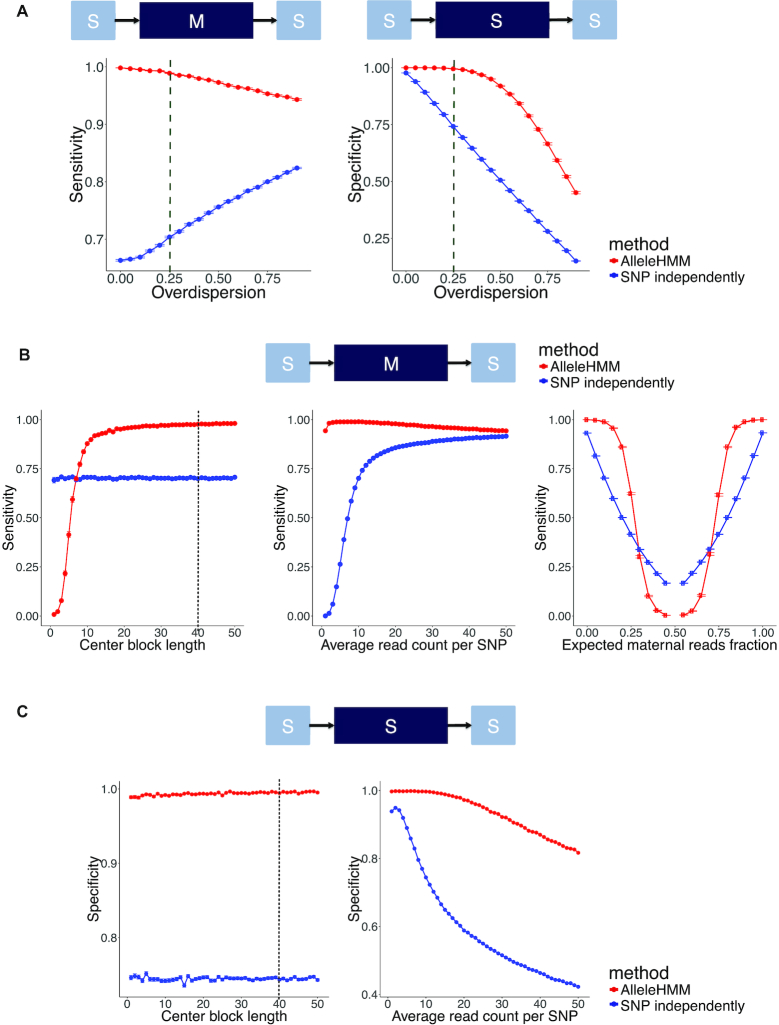
AlleleHMM had better sensitivity and specificity compared with the naive standard practice of performing a binomial test for each SNP independently in overdispersed data. (**A**) Scatterplots show sensitivity (left) and specificity (right) of AlleleHMM (red) and independent binomial tests (blue) as a function of the overdispersion parameter in beta-binomial distributed simulated data. Error bars represent the standard error of 1000 independent simulations. Dashed lines indicate the mean of overdispersion estimated from GRO-seq of GM12878 and GRO-seq of 129/*castaneus* F1 hybrid mESCs. (**B**) Scatterplots show the sensitivity of AlleleHMM (red) and independent binomial tests (blue) as a function of the length of a maternal specific blocks (the number of continuous SNPs sharing same allele specificity, left), the average read count at each SNP (center), or the expected maternal read fraction (right) with an overdispersion of 0.25. Error bars represent the standard error of 1000 independent simulations. The dotted line indicates the average number of SNPs per human gene. (**C**) Scatterplots show the specificity of AlleleHMM (red) and independent binomial tests (blue) as a function of the length of the symmetric middle block (left) or the average read count at each SNP (right) with an overdispersion of 0.25. Error bars represent the standard error of 1000 independent simulations. The dotted line indicates the average number of SNPs per human gene.

Many short-read datasets exhibit overdispersion due to a variety of technical factors, which increases the rate of false positive allele specific differences (5). To test how AlleleHMM performed with overdispersed data, we applied a similar simulation strategy using a beta-binomial distribution to simulate read counts with varying degrees of overdispersion. AlleleHMM had a reasonably high sensitivity, precision, and specificity across the spectrum of distinct overdispersion values (Figure 3A; Supplementary Figure S3A). AlleleHMM retained a sensitivity >0.95, while maintaining both precision and specificity near 1.0 at realistic overdispersion levels estimated using two independent GRO-seq datasets: human GM12878 lymphoblastoid cells (overdispersion of 0.24) (18) and 129/*castaneus* F1 hybrid mouse embryonic stem cells (mESCs) (overdispersion of 0.26) (19).

To test how AlleleHMM performance varied with the length, signal level, and the degree of allele specificity when the input data was overdispersed, we fixed overdispersion to 0.25 (dashed lines in Figure 3A and Supplementary Figure S3A) and performed simulation experiments similar to those described for the binomial distribution, above. AlleleHMM sensitivity and precision increased with block length, and were higher than independent binomial tests with as few as 8 (or 5, respectively) adjacent SNPs (Figure 3B, Supplementary Figure S3B, left). AlleleHMM was also highly sensitive over an important range of allele specificity magnitudes (≤0.25 or ≥0.75; Figure 3B, right). AlleleHMM had a higher specificity than independent binomial tests across the spectrum of length (the number of SNPs per gene, Figure 3C, left) and signal levels (average read counts per SNP, Figure 3C, right). The specificity of both AlleleHMM and independent binomial tests declined as read count increased (Figure 3C, right). However, AlleleHMM maintained a reasonable precision throughout this range, while independent binomial tests never achieved a precision >0.75 in this test (Supplementary Figure S3, center). Moreover, AlleleHMM exhibited a high sensitivity within the range at which it maintained a high specificity (2–20 reads supporting each SNP, Figure 3B, center), suggesting that subsampling highly expressed regions may be a viable strategy to deal with overdispersion in practice. Thus, AlleleHMM improved specificity in overdispersed data while maintaining a higher sensitivity compared with state-of-the-art tools using realistic parameters taken from GRO-seq data.

### Performance comparison with independent SNPs using GRO-seq data

We next asked whether AlleleHMM provides a higher sensitivity, specificity, or precision using real GRO-seq data as input. We used AlleleHMM to analyze two public GRO-seq datasets: one from 129/*castaneus* F1 hybrid mESCs and the other from a human GM12878 lymphoblastoid cell line (18,19).

**Figure 4.**
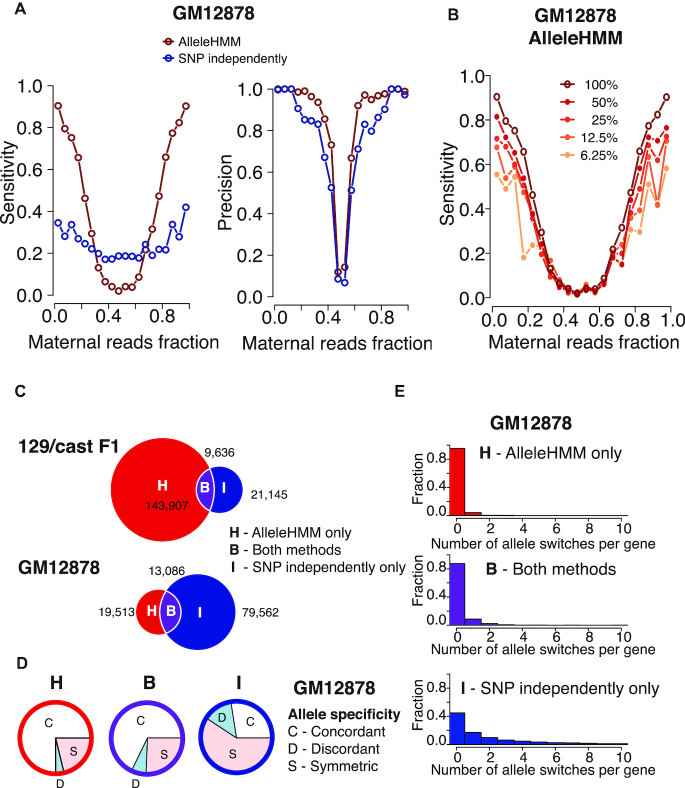
Comparison between AlleleHMM and independent binomial tests. (**A**) Scatterplots show the sensitivity (left) and precision (right) of AlleleHMM (red) and independent binomial tests (blue) as a function of the maternal reads fraction in the gene annotation using GRO-seq data from GM12878. (**B**) Scatterplots show the sensitivity of AlleleHMM as a function of the fraction of maternal reads in the gene annotations. Different lines indicate the read depth of the subsampled GRO-seq reads from a deeply sequenced human GM12878 dataset. The total sequencing depth is 187 896 441. (**C**) Venn diagrams show the number of allele specific SNPs identified by AlleleHMM only (H, red), independent binomial tests (I, blue, implemented in AlleleDB), and intersection of both methods (B, purple) from GRO-seq data of a 129/*castaneus* F1 hybrid mouse (top) and a human cell line GM12878 (bottom). (**D**) Pie charts show the proportion of allele specific SNPs in GM12878 GRO-seq data that are within genes having no evidence of allele specificity over the gene (symmetric, S, pink), or genes that show higher expression on the same (concordant, C, white) or the opposite (discordant, D, light blue) haplotype. (**E**) Histograms show the fraction of genes as a function of the number of allele specificity switches the gene contains. Allele specificity was determined by AlleleHMM only (H, red, top), independent binomial tests (I, blue, bottom), and intersection of both methods (B, purple, middle) using GRO-seq data from a human cell line GM12878.

To estimate the accuracy of AlleleHMM using real data in which there was no ground truth, we identified annotated genes with evidence for allele specific transcription by combining reads across the entire gene annotation. Because RNA polymerase in the gene body is loaded in the promoter region, SNPs residing in the same gene annotation should generally share the same level of allele specificity. Therefore we estimated the allele specificity of each gene annotation using all reads that fall inside, and assigned this magnitude of allele specificity to every SNP that resides inside that annotation (see Methods). Using gene annotations as a surrogate for a ground truth, we found that AlleleHMM had a higher sensitivity than independent binomial tests, especially within the range of allele specificity magnitudes that was most likely to contain biologically relevant allelic differences (maternal read ratio of the gene ≤0.25 or ≥0.75, Figure 4A, Supplementary Figure S4, left), consistent with its performance in synthetic data. AlleleHMM had a similar or higher precision compared with independent binomial tests (Figure 4A, Supplementary Figure S4, right). Thus, AlleleHMM is both more sensitive and precise when using real GRO-seq data as input.

Most genomics applications are highly imbalanced, with negative examples greatly outnumbering positive examples, resulting in poor precision despite a high specificity. To measure performance in cases where data are highly imbalanced, we generated datasets based on SNPs in genes where the magnitude of allele specificity was <0.2 or >0.8 (true positives) or between >0.45 and <0.55 (true negatives). Using these highly imbalanced datasets (ratio of positive to negative examples < 0.05), AlleleHMM retained a high precision (>0.66) at relatively high sensitivity (>0.76). AlleleHMM outperformed independent binomial tests by a wide margin in this task (sensitivity < 0.4 and precision < 0.2; see Supplementary Figure S5). We note that a precision of 1.0 is not necessarily expected or desired in these tests, because numerous differences exist between gene annotations and the actual patterns of transcription that occur in cells (as described below). These results suggest that even in a setting where less than 5% of SNPs are true positives, AlleleHMM still obtains useful information about allele specificity, whereas the use of independent tests does not.

To examine how library sequencing depth affects the performance of AlleleHMM, we subsampled GRO-seq reads from the deeply sequenced human GM12878 dataset and evaluated recovery using annotations with true positive/ negative labels generated based on the entire dataset. We found that AlleleHMM was remarkably sensitive even at sequencing depths that were <10% of the total read count (Figure 4B), at a consistent precision and specificity (Supplementary Figure S6A, center and right). In contrast, the sensitivity of independent binomial tests, which was not very high to begin with, dropped off rapidly with sequencing depth (Supplementary Figure S6B, left). Thus, even a modest sequencing depth (∼20 million uniquely mapped reads) is enough to identify the majority of allele specific differences in transcription using GRO-seq data.

To more rigorously understand the differences between AlleleHMM and independent binomial tests, we divided SNPs based on whether they were identified as allele specific using AlleleHMM, independent binomial tests, or both methods. Few SNPs were identified as allele specific using both AlleleHMM and independent binomial tests on each SNP (Figure 4C). In GM12878, for example, AlleleHMM identified 32,599 heterozygous SNPs with 1 or more read in 4,026 AlleleHMM blocks. Only 13,086 of the SNPs identified using AlleleHMM were also discovered using independent binomial tests (∼40% of SNPs; Figure 4C, bottom). As expected, SNPs identified only by AlleleHMM largely reflect heterozygous positions covered by too few reads to confidently assign allele specificity when treating SNPs independently, whereas those identified using both methods had a higher read depth (Supplementary Figure S7). Taken together, these observations are consistent with AlleleHMM making substantial improvements in sensitivity for allele specific differences in genes with lower expression levels.

We were more surprised to find large numbers of SNPs reported as allele specific using independent binomial tests without a corresponding discovery by AlleleHMM (*n* = 21,145 [mESC] or 79,562 [GM12878]). To investigate whether these SNPs were false negative calls by AlleleHMM or false positives by the binomial test, we again used SNPs within annotated genes under the assumption that RNA polymerase across a gene shares the same allele specificity. The majority of allele specific SNPs identified by AlleleHMM were found to have the same direction (maternal or paternal) of allele specificity as the gene annotation, henceforth called ‘concordant’ (Figure 4D, concordant [C] in white). By contrast, SNPs identified as allele specific using only independent binomial tests were most often identified within genes where the entire annotation showed no evidence of allele specificity, henceforth called ‘symmetric’ (Figure 4D, symmetric [S] in pink). We also directly investigated whether gene annotations tend to have a single allelic state using each method, as implied by the assumption that RNA polymerase density is largely determined by events occurring at a single promoter region. We found that AlleleHMM identified a single block covering annotations in >80% of cases (Figure 4E, top). In contrast, the direction of allele specificity detected by independent binomial tests often switched across the annotation (Figure 4E, bottom), resulting in no evidence of allele specificity when SNPs within annotations were merged. Taken together, these observations provide additional support to our analysis of precision in unbalanced genomic data (Supplementary Figure S5) and suggest that many of the SNPs identified only by independent binomial tests are false positives.

### Widespread non-coding allele specific transcription identified using AlleleHMM

**Figure 5.**
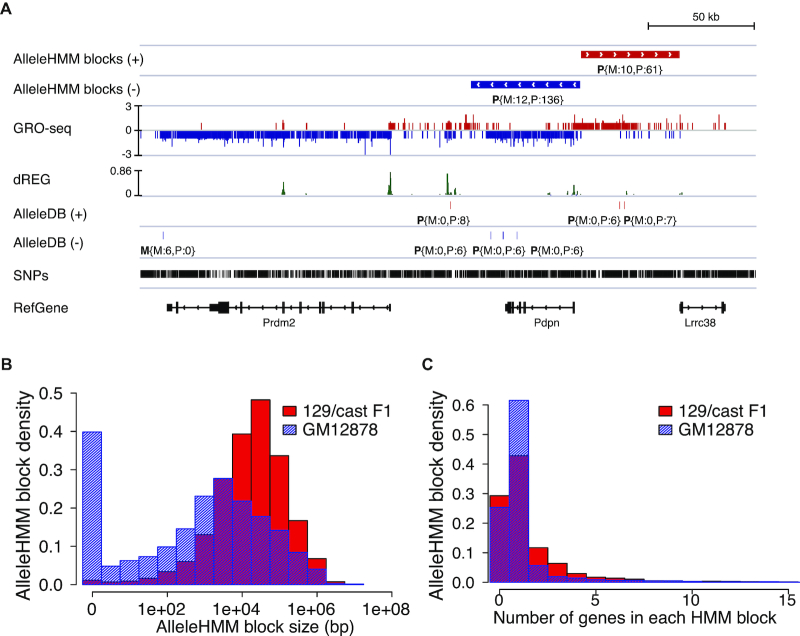
Application of AlleleHMM to GRO-seq data. (**A**) Genome browser view shows the application of AlleleHMM and independent binomial tests (implemented in AlleleDB) to GRO-seq data from a 129/*castaneus* F1 hybrid mouse. The allele specific read counts of the blocks and SNPs are denoted as **P**{M:12,P:136}, meaning that the block is paternal specific (P) with 12 maternal-specific (M) reads and 136 paternal-specific (P) reads. (**B**) Histograms show the distribution of AlleleHMM block size of GRO-seq data from a 129/*castaneus* F1 hybrid mouse (red) and GRO-seq data from a human cell line GM12878 (blue) in log scale (X-axis). (**C**) Histograms show the fraction of AlleleHMM blocks as a function of the number of genes it contains. GRO-seq data from a 129/*castaneus* F1 hybrid mouse is in red and GRO-seq data from a human cell line GM12878 is in blue.

Despite achieving a high concordance with gene annotations when available, AlleleHMM was also able to identify allele specific differences in unannotated transcription units. For instance, AlleleHMM identified the transcription unit upstream and antisense to *Pdpn* as sharing the same allele specificity as the *Pdpn* coding region (Figure 5A). Likewise, in cases where AlleleHMM disagreed with gene annotations, it frequently identified cell-type specific transcription units that were correctly classified upon careful examination. For instance, in the GM12878 dataset AlleleHMM found that transcription originating from enhancers within the *DTNB* gene annotation was maternal-specific, although most of the *DTNB* annotation itself was not (Supplementary Figure S8). These observations demonstrate that AlleleHMM provides substantial advantages useful for new biological discovery compared with heuristics that summarize signals within well-known gene annotations.

AlleleHMM revealed thousands of regions with maternal or paternal-specific RNA polymerase abundance genome-wide. AlleleHMM identified 3,483 and 4,026 blocks with significant allele specific differences in mESCs and GM12878, respectively. The average genome size of each block in the F1 hybrid dataset was ∼166 kb (Figure 5B). Blocks were larger in the mESC dataset than in GM12878, likely owing to a combination of differences in heterozygosity and sequencing depth between datasets (Supplementary Figure S9). Approximately 25% of AlleleHMM blocks did not contain any GENCODE gene annotation (Figure 5C), for example the antisense transcription unit upstream of *Pdpn* (Figure 5A). Many AlleleHMM blocks contained more than one gene annotation (28% of F1 hybrid mouse blocks and 13% of GM12878 blocks), indicating groups of nearby genes and non-coding transcription units that share similar allele specificity in their transcription. Thus, AlleleHMM identified widespread evidence for allele specificity in non-coding transcription, as well as coordination between nearby transcription units that was not evident from strategies that merged gene annotations.

### Allele specific transcription negatively correlates with allele specific repressive chromatin marks

**Figure 6.**
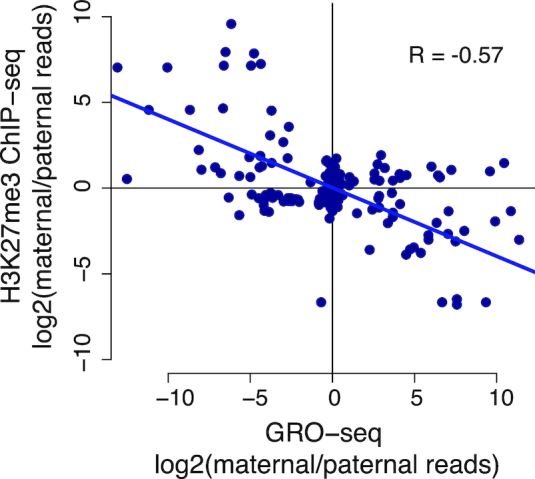
Allele specific transcription correlates with allele specific H3K27me3. Scatterplots show the allele specificity of H3K27me3 ChIP-seq data (Y-axis) as a function of allele specificity in GRO-seq data (X-axis). The trendline shows the best fit based on a total least squares regression. The Pearson correlation is shown on the plot (*R* = –0.57, *P*-value < 2.2e–16). The slope of the best fit line is –2.5.

To find further independent validation for blocks of allele specific transcription identified using AlleleHMM, we asked whether we could recover the negative relationship expected between transcription and histone marks associated with transcriptional repression, especially H3K27me3. We focused on GM12878, for which there is publicly available ChIP-seq data profiling the distribution of H3K27me3 (20,21). Mapping H3K27me3 ChIP-seq data onto AlleleHMM blocks identified using GRO-seq revealed 113 blocks with a significant allele specificity in H3K27me3 ChIP-seq data. As expected, the degree of allele specificity in H3K27me3 ChIP-seq was inversely correlated with that of GRO-seq (Pearson's *R* = –0.57; Figure 6). The slope of the best fit line implies that a 2-fold change in H3K27me3 was associated with ∼5.7-fold change in transcription. Assuming a similar dynamic range in both assays, this result implies that relatively modest changes in H3K27me3 may have a relatively large average impact on transcription. Thus, AlleleHMM reveals blocks of allele specificity which are largely in agreement with orthogonal genomic assays.

## DISCUSSION

Here we have introduced AlleleHMM to identify allele specific differences in functional genomic marks. AlleleHMM models the spatial correlation in allele specific differences in a phased diploid genome using hidden Markov models (HMMs). We show that AlleleHMM provides substantial improvements in both sensitivity and specificity for detecting allele specific SNPs compared with existing computational tools, using both simulation studies and analyses of real GRO-seq data. AlleleHMM is applicable to any type of functional genomic data and to any diploid species with a high-quality phased reference genome. AlleleHMM can now be deployed to understand the interplay between chromatin environment, transcription, and mRNA across a wide variety of organisms, providing new insights into how DNA sequences influence biochemical processes in the nucleus.

Although there has been extensive interest in using allele specific information to understand the interplay between functional marks, surprisingly few computational methods have been developed for this task. One of the current methods to identify allelic differences requires performing independent statistical tests at each SNP (4,5). This approach of treating SNPs independently requires a high sequencing depth with dozens of reads covering each SNP in order to be statistically powered to identify allele specific differences. Additionally, because signal for the majority of marks tends to be unevenly distributed along the genome, this strategy is prone to significant biases where loci with a higher signal intensity tend to be better represented due to statistical power. Finally, as has been reported elsewhere (5), and as we show here, the application of independent statistical tests is prone to a high rate of false discoveries. Although false positives can be addressed by using more conservative statistical models (5), this more conservative strategy exacerbates issues with statistical power. AlleleHMM addresses this deficiency in an alternative way, by modelling the correlation between adjacent signal intensities, thus pooling statistical power across adjacent positions.

An alternative approach that is commonly used to identify allele specific differences in mark abundance is to use pre-established boundaries of genes or other genomic features as a way to pool SNPs within regions of the genome (6). This alternative strategy improves upon the use of independent statistical tests by using information between nearby alleles. However, there are still a number of important limitations with this approach. Chiefly among the limitations of this strategy is that allele specific differences in functional marks cannot be identified if they fall outside of the boundaries of pre-established gene annotations. Likewise, cell-type specific differences in transcript isoforms are common, even in well annotated genomes like human and mouse, which provide a substantial source of error for annotation-based approaches. Finally, the use of annotations requires a well annotated reference genome, which is only available in well studied model organism such as Drosophila, humans, or mice. AlleleHMM addresses these limitations by providing a rigorous and statistically motivated method to identify the boundaries of allele specific blocks *de novo*.

Although AlleleHMM is a powerful tool that makes significant improvements compared with existing strategies, it does have several limitations. Chiefly among these, AlleleHMM will provide the most significant benefit for functional assays where marks are spread broadly across the genome, rather than focused within specific functional regions. This provides the broadest benefit for assays such as GRO-seq, RNA-seq, and several of the broadly distributed ChIP-seq marks. AlleleHMM may provide less benefit for assays such as 3′ mRNA-seq or chromatin accessibility assays (e.g., ATAC-seq or DNase-I-seq), where signals are distributed within a specific position of the genome. Nevertheless, AlleleHMM will still work under these settings, and may still provide a substantial benefit for detecting expression differences that span multiple genes or chromatin accessible regions.

Using AlleleHMM, we have identified thousands of regions harboring allele specific differences in human GM12878 and murine ESCs. We have found that allelic differences tend to occur over large genomic regions that harbor multiple transcription units, often sharing the same gene annotation. This finding is reminiscent of the shared architecture of quantitative trait loci (QTLs) across broad genomic regions (22). This finding may also reflect similar regulatory principles as the positionally dependent variation in gene expression across distinct biological replicates (23). Altogether, the use of AlleleHMM provides a novel tool that will be useful to rigorously examine how homologous DNA sequences in the nucleus differ in the distribution of functional genomic marks. We are confident that future studies will use this tool to unravel multiple aspects of genome function and organization.

## Supplementary Material

gkz176_Supplemental_File
