# Supplementary material for: AlleleHMM: a data-driven method to identify allele specific differences in distributed functional genomic marks
Source: Nucleic Acids Res. 2019 Mar 28;47(11):e64. doi: 10.1093/nar/gkz176 (PMC6582321; doi:10.1093/nar/gkz176)
Supplement: gkz176_Supplemental_File [file gkz176_supplemental_file.pdf]

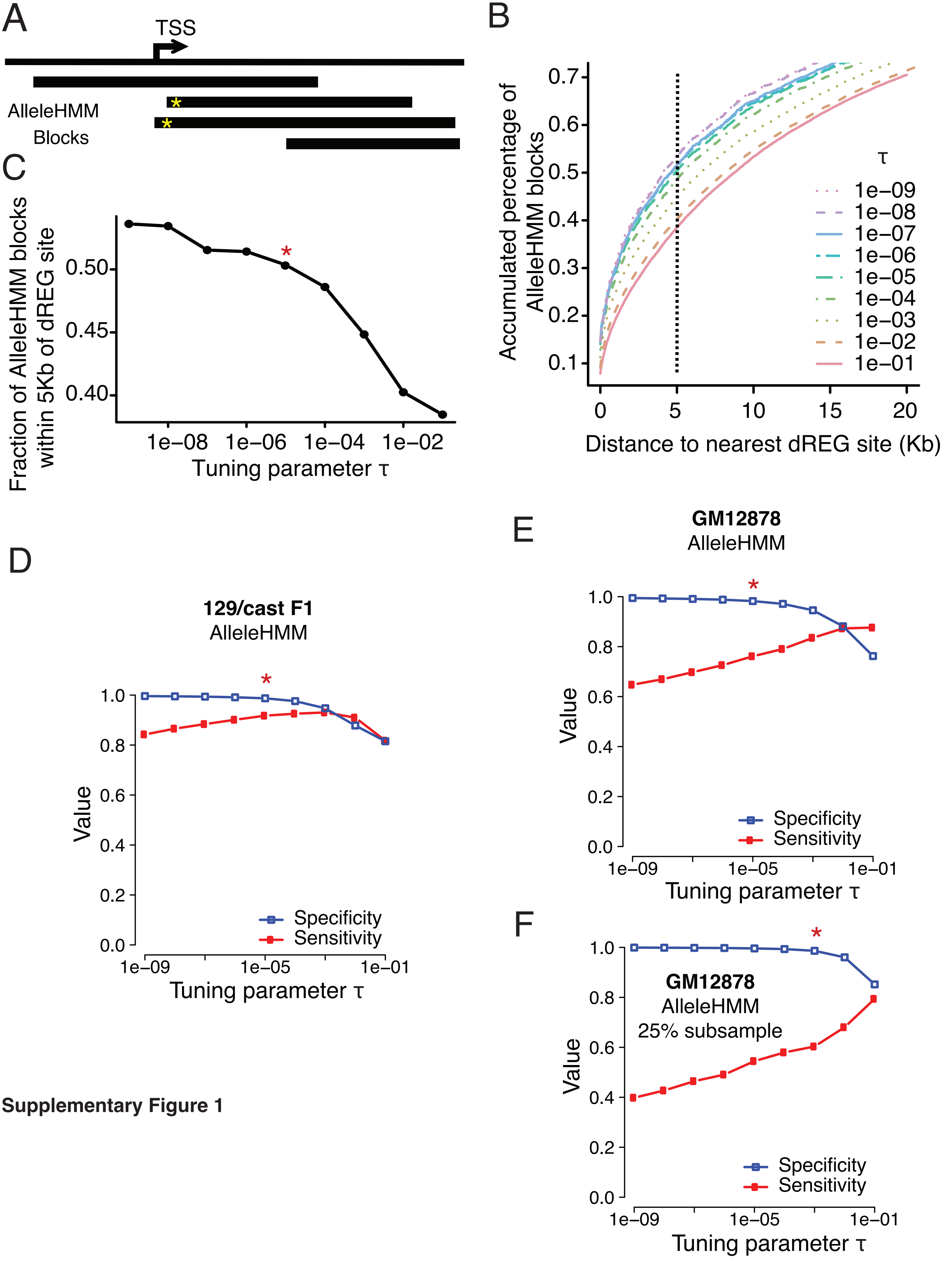

Supplementary Figure 1

Supplementary Figure 1: Selection of values for the tuning parameter used to control the balance between AlleleHMM sensitivity and specificity.

(A) Cartoon illustrates how we set the tuning parameter τ. We assumed that SNPs within the same transcript have a similar allele specificity. Therefore, the optimum value of τ should approach a saturation point to maximize the fraction of state transitions near a transcription start site (TSSs) that is active in that cell type. The black bars are AlleleHMM blocks, represents a region with significant allele specificity. Those with a yellow star have state transitions near TSSs.

(B) Plot shows the distance between the beginning of AlleleHMM blocks and its closest TSS identified using dREG for GRO-seq data from a 129/*castaneus* F1 hybrid mouse. Different lines indicate AlleleHMM blocks predicted using different values of the tuning parameter, τ.

(C) Scatterplot shows the fraction of AlleleHMM blocks within 5 kb of the nearest TSS predicted by dREG (Y-axis) as a function of the tuning parameter τ (X-axis) used for GRO-seq data from a 129/*castaneus* F1 hybrid mouse. The red star indicates a value near a point of saturation (τ = 1e-5) used for the remainder of this study.

(D) Scatterplots show the sensitivity (red) and specificity (blue) of AlleleHMM as a function of the tuning parameter τ (X-axis) used for GRO-seq data from a 129/*castaneus* F1 hybrid mouse. The red star indicates the value of τ (1e-5) used for the remainder of this study.

(E) Scatterplots show the sensitivity (red) and specificity (blue) of AlleleHMM as a function of the tuning parameter τ (X-axis) used for GRO-seq data from GM12878. The red star indicates the value of τ (1e-5) used for the remainder of this study.

(F) Scatterplots show the sensitivity (red) and specificity (blue) of AlleleHMM as a function of the tuning parameter τ (X-axis) used for a 25% subsampled GRO-seq data from GM12878. The red star indicates the value of τ (1e-3) used for the remainder of this study.

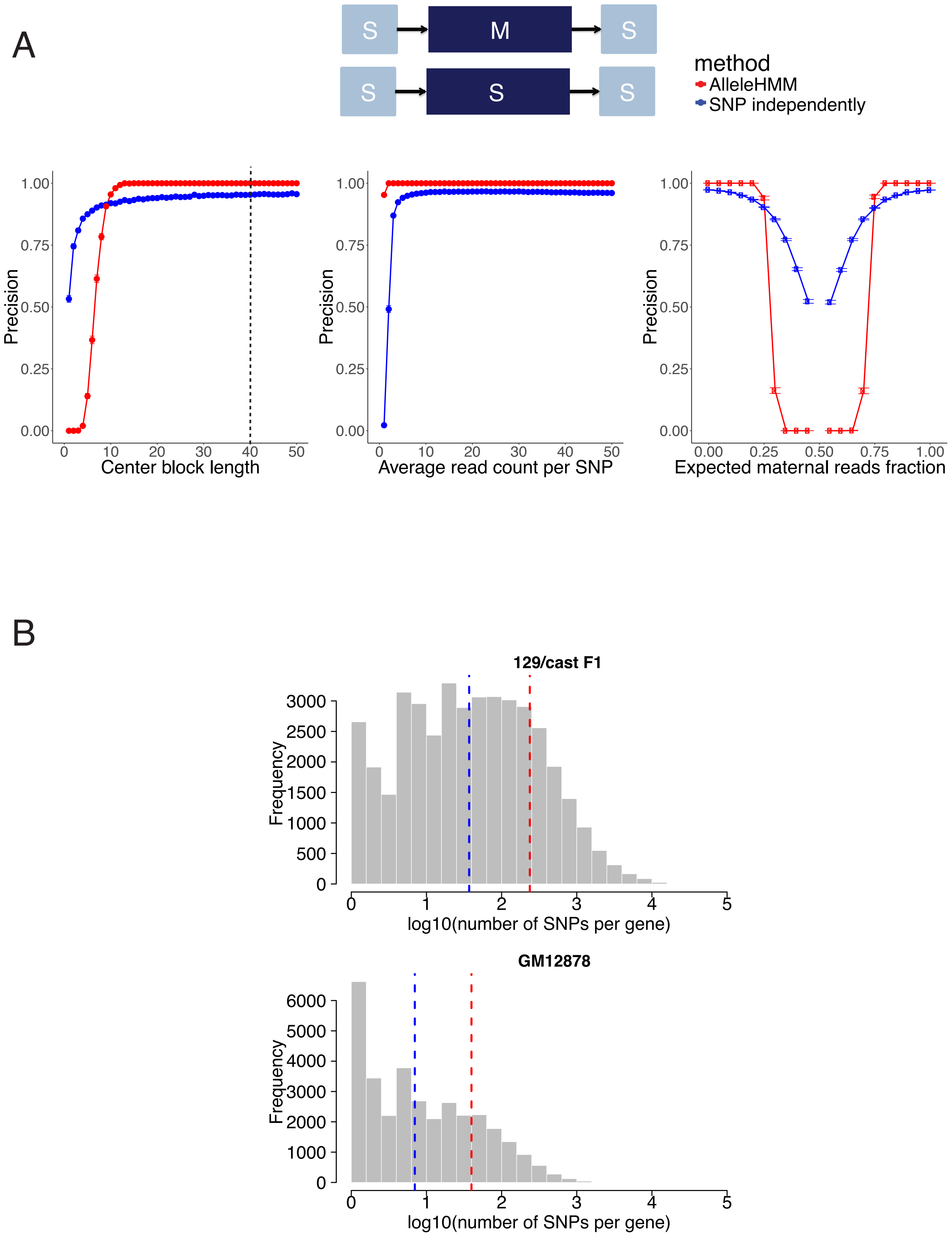

Supplementary Figure 2

Supplementary Figure 2: AlleleHMM had better precision compared with standard methods performing independent binomial tests for each SNP.

(A) Scatterplots show the precision for each SNP in the center block of AlleleHMM (red) and independent binomial tests (blue) as a function of the length of the center block (the number of continuous SNPs sharing same allele specificity, left), the average read count at each SNP (center), or the expected maternal reads fraction (right). Error bars represent the standard error of 1000 independent simulations. The dotted line indicates the average number of SNPs per human gene.

(B) Histograms show the distribution of the number of SNPs per gene in a 129/*castaneus* F1 hybrid mouse (top) and a human cell line GM12878 (bottom) in log scale (X-axis). Blue dashed lines indicate the median of the number of SNPs per gene (129/*cast* is 37.0, GM12878 is 7.0) and red dashed lines indicate the mean (129/*cast* is 237.2, GM12878 is 39.7).

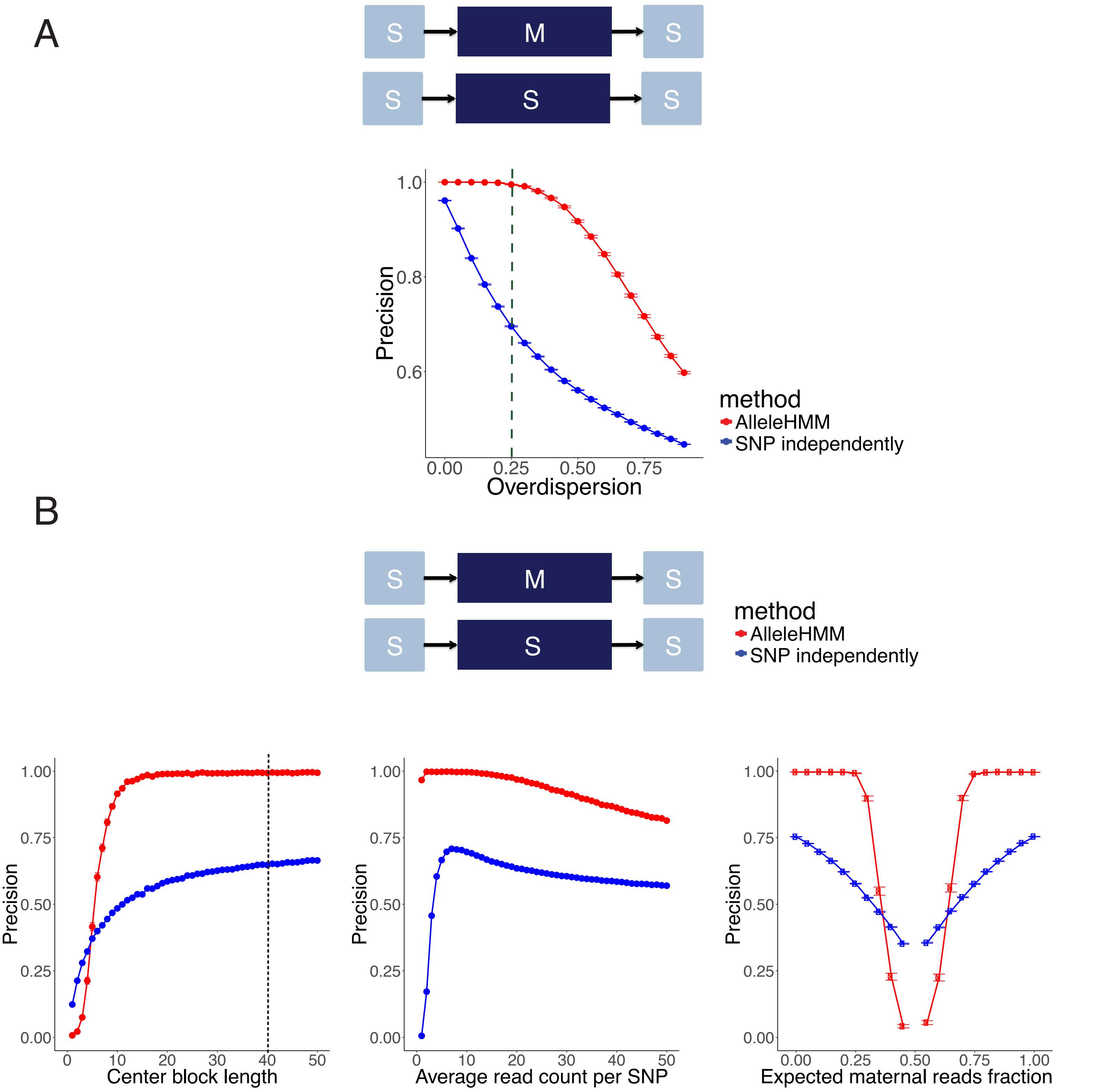

Supplementary Figure 3: AlleleHMM had better precision compared with standard methods performing independent binomial tests for each SNP in overdispersed data.
(A) Scatterplots show precision for each SNP in the center block of AlleleHMM (red) and independent binomial tests (blue) as a function of the overdispersion parameter in beta-binomial distributed simulated data. Error bars represent the standard error of 1000 independent simulations. Dashed lines indicate the mean of overdispersion estimated from GRO-seq of GM12878 and GRO-seq of 129/*castaneus* F1 hybrid mESCs.
(B) Scatterplots show the precision for each SNP in the center block of AlleleHMM (red) and independent binomial tests (blue) as a function of the length of the center block (the number of continuous SNPs sharing same allele specificity, left), the average read count at each SNP (center), or the expected maternal reads fraction (right) with an overdispersion of 0.25. Error bars represent the standard error of 1000 independent simulations. The dotted line indicates an estimate number of SNPs per human gene.

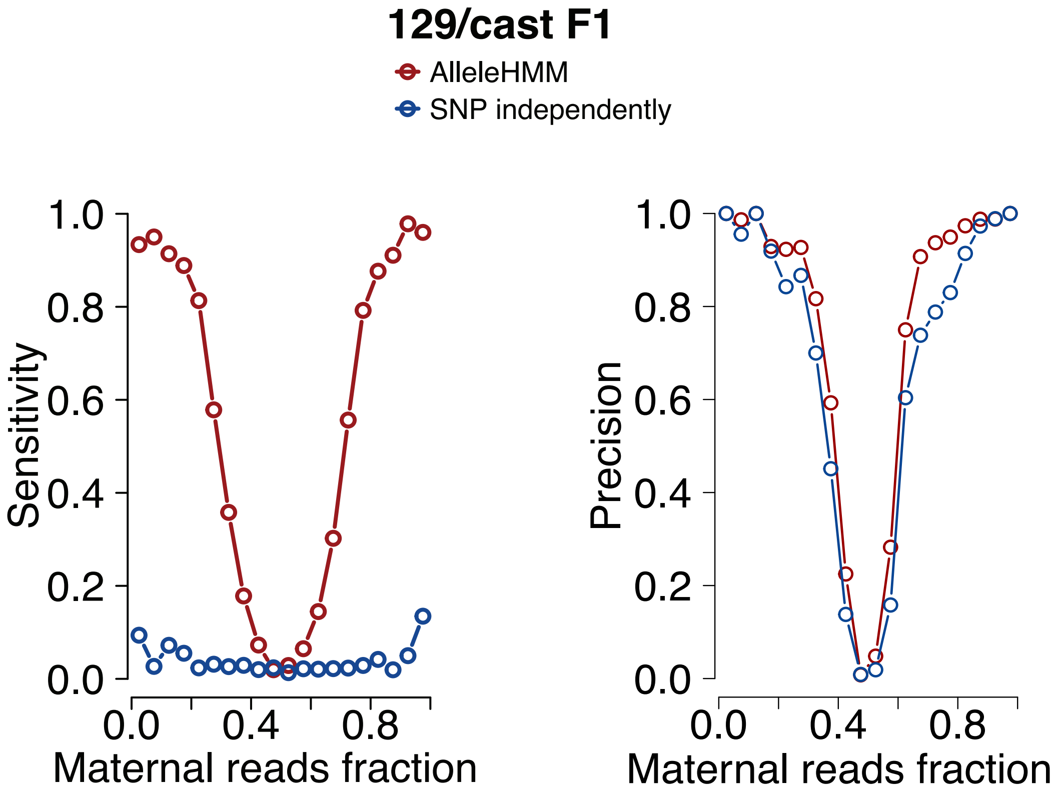

Supplementary Figure 4: Comparison of sensitivity and precision using GRO-seq data. Scatterplots show the sensitivity (left) and precision (right) of AlleleHMM (red) and independent binomial tests (blue) as a function of the maternal reads fraction in the gene annotation using GRO-seq of 129/*castaneus* F1 hybrid mESCs.

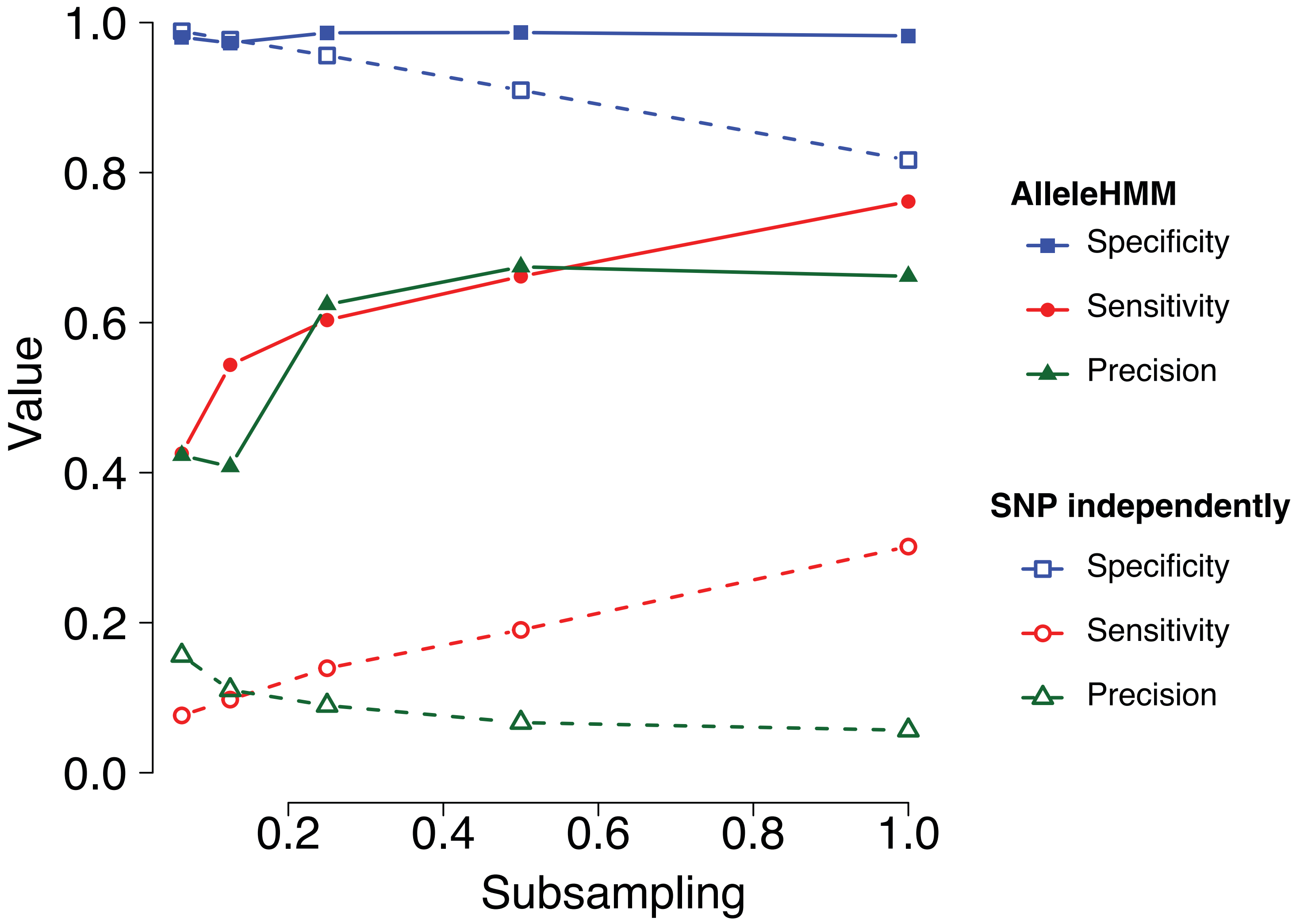

Supplementary Figure 5: Scatterplot shows the specificity (blue), sensitivity (red), and precision (green) of AlleleHMM (filled symbol) and independent binomial tests (unfilled symbol) as a function of read depth (subsampled from a total dataset) in human GM12878 GRO-seq data. The values were estimated using SNPs in genes defined as symmetric or allele specific. Symmetric genes have a maternal reads fraction between 0.45 and 0.55 and was classified as symmetric using all the mapped reads in the gene annotation (FDR > 0.1). Allele specific genes have maternal read fractions <0.2 or >0.8 and was classified as significantly allele specific (FDR < 0.01) by performing a binomial test using all the reads in the gene.

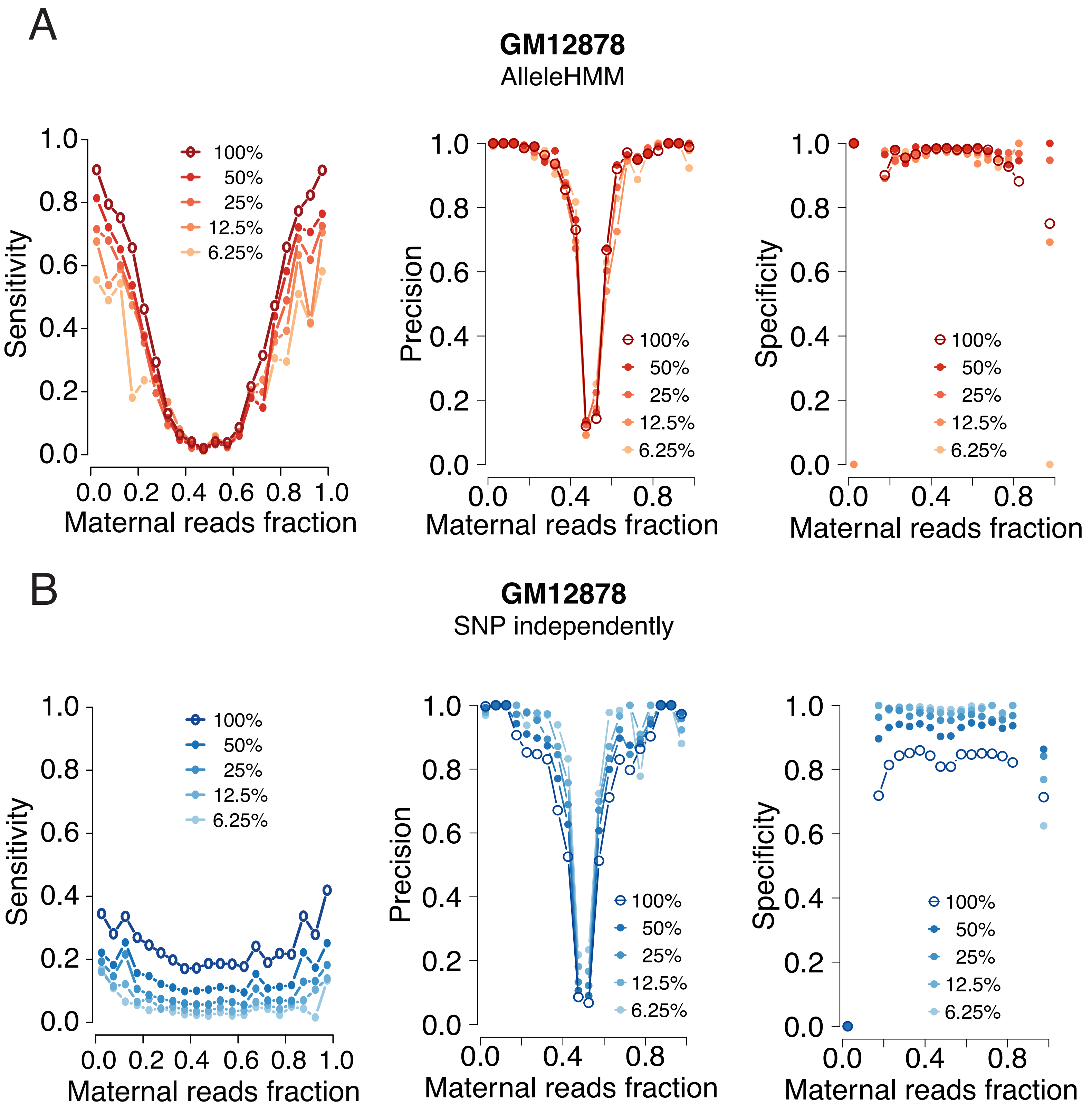

Supplementary Figure 6: Effects of subsampling GM12878 on sensitivity, precision, and specificity. (A) Scatterplots show the sensitivity (left), precision (center), and specificity (right) of AlleleHMM as a function of the maternal reads fraction in the gene annotation. Different lines indicate the read depth of the subsampled GRO-seq reads from a deeply sequenced human GM12878 dataset. The total sequencing depth at 100% is 138 millions uniquely mapped reads.

(B) Scatterplots show the sensitivity (left), precision (center), and specificity (right) of independent binomial tests as a function of the maternal reads fraction in the gene annotation. Different lines indicate the read depth of the subsampled GRO-seq reads from a deeply sequenced human GM12878 dataset.

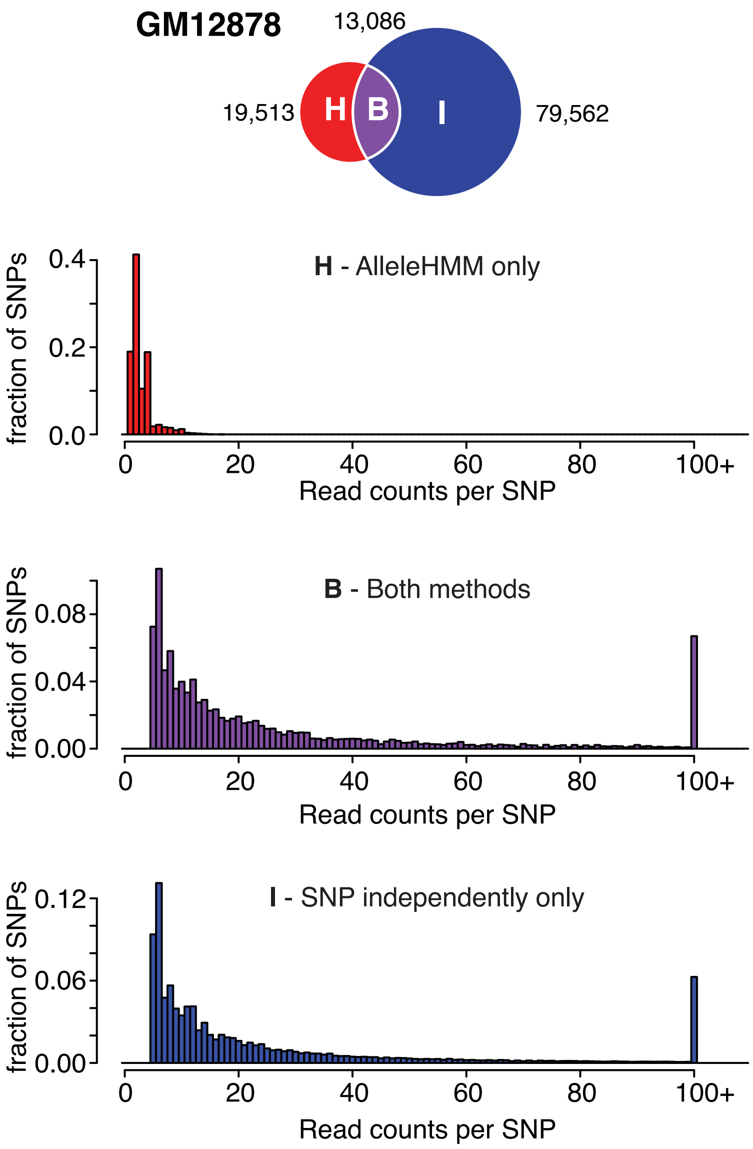

Supplementary Figure 7: AlleleHMM identifies blocks with fewer reads supporting each SNP. Histograms show the fraction of SNPs as a function of the read counts per allele specific SNP identified by AlleleHMM only (H, red, top), independent binomial tests (**I**, blue, bottom), and the intersect of both methods (B, purple, middle) using GRO-seq data from GM12878.

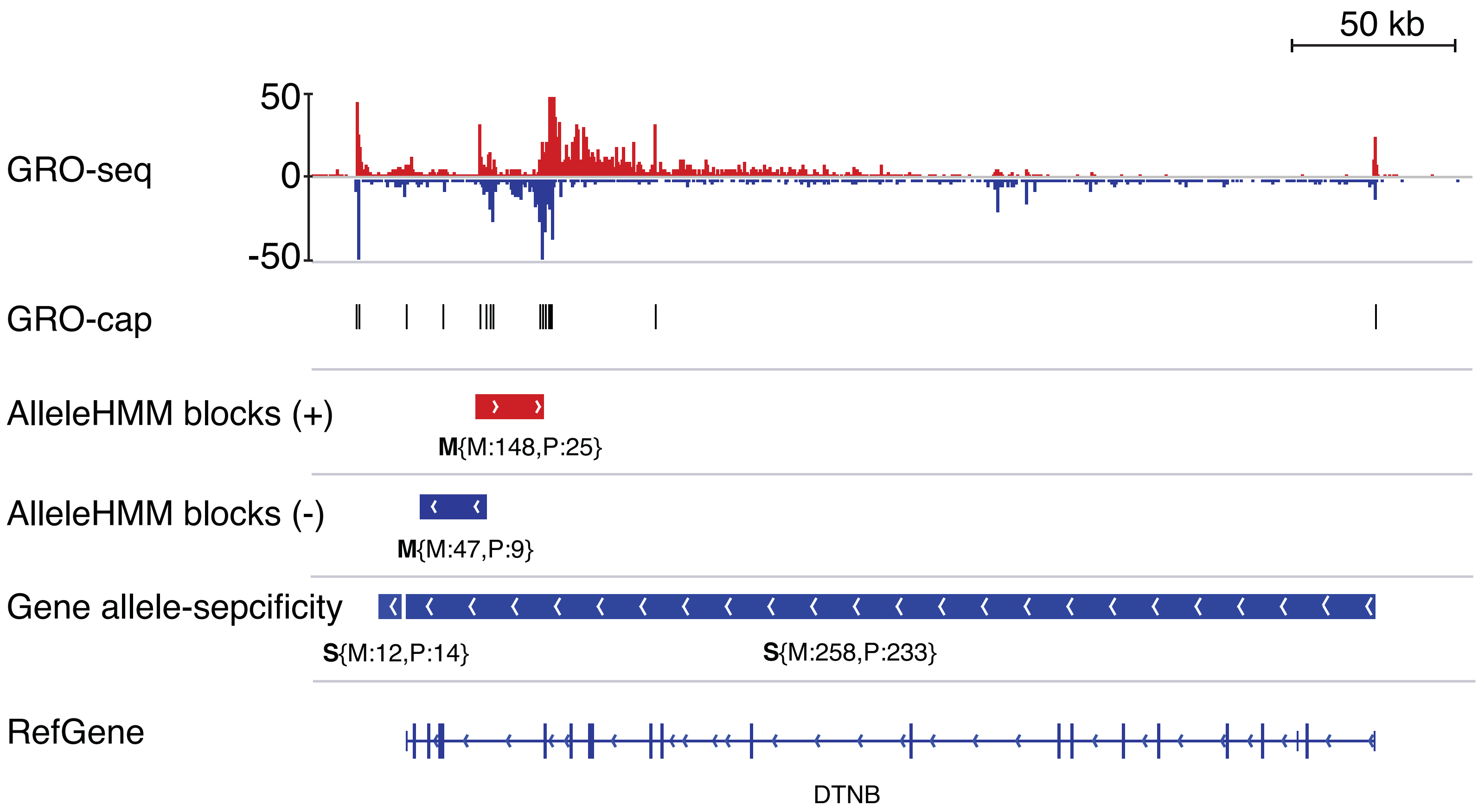

Supplementary Figure 8: Genome browser view shows the application of AlleleHMM to GRO-seq data from a human cell line GM12878. The allele specific read counts of the blocks are denoted as **S**{M:258,P:233}, meaning that the block is symmetric (S) with 258 maternal-specific (M) reads and 233 paternal-specific (P) reads. GRO-cap data denotes transcription start sites obtained from ref (18).

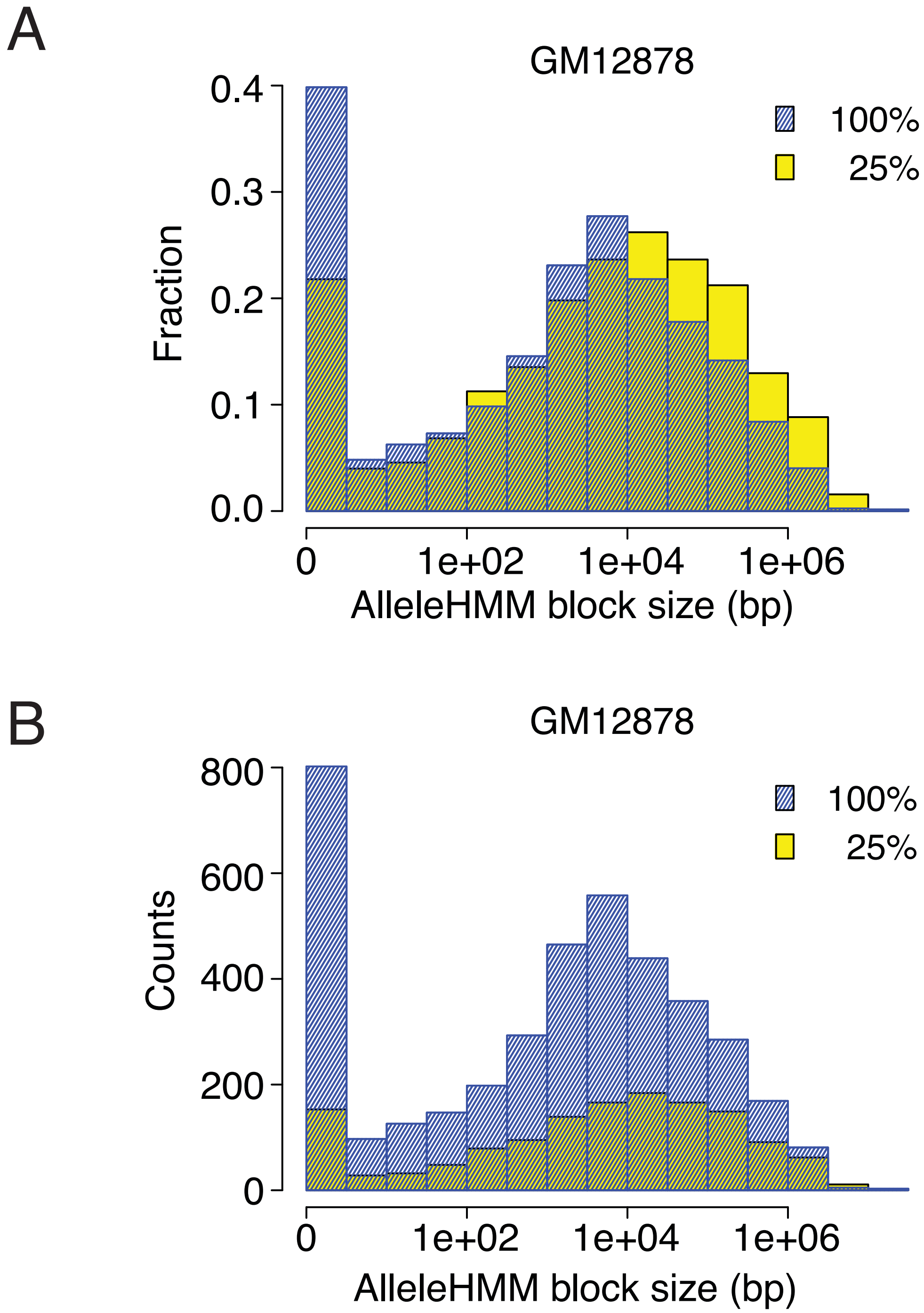

Supplementary Figure 9: The size of AlleleHMM blocks increased as read depth decreased. Histograms show the fraction of AlleleHMM blocks having a block size indicated on the X axis in log scale. Data is shown for the full GM12878 GRO-seq dataset (187,896,441 reads; blue), or a mock dataset with 25% subsample (46,974,111 reads ;yellow).
Histograms show the counts of AlleleHMM blocks as a function the block size in log scale (X-axis). The blue histogram was calculated using total GRO-seq reads (187,896,441) from GM12878, yellow was calculated using a 25% subsample dataset of 46,974,111 reads.

Supplementary Table 1: An example of input file for AlleleHMM.

chrm	snppos	mat_allele_count	pat_allele_count
chr1	565006	0	17
chr1	565286	46	0
chr1	565406	37	0
chr1	565419	31	0
chr1	565591	27	0
chr1	566573	0	2
chr1	568214	0	6
chr1	569094	93	0
chr1	569933	0	2
chr1	724882	2	0
chr1	724883	2	0
chr1	726939	1	4
chr1	726944	5	0
chr1	940005	1	0
[…]			
chr22	50982539	0	1
chr22	50989326	0	2
chr22	51010052	2	0
chr22	51017353	2	0
chr22	51023408	0	2
chr22	51023424	0	2
chr22	51059835	1	2
chr22	51066921	0	2
chr22	51189146	0	1
chr22	51222766	0	1

Supplementary Table 2: Parameters used in the synthetic data.

**Figures**	Y-axis **Sensitivity, Precision**	**Number of SNPs per block**			**Average read count per SNP**			**Expected maternal reads fraction**		
	X-axis	S	M	S	S	M	S	S	M	S
Figure2 A, left; Supplementary Figure2A, left	Center block length	10	**1, 2, …, 50**	10	10	10	10	0.5	0.9	0.5
Figure2A, center; Supplementary Figure2A, center	Average read count per SNP	10	100	10	10	**1, 2, …, 50**	10	0.5	0.9	0.5
Figure2A, right; Supplementary Figure2A, right	Expected maternal reads fraction	10	100	10	10	10	10	0.5	**0, 0.05, 0.10, …, 1**	0.5
	Y-axis **Specificity, Precision**									
	X-axis	S	S	S	S	S	S	S	S	S
Figure2B, left	Center block length	10	**1, 2, …, 50**	10	10	10	10	0.5	0.5	0.5
Figure2B, right	Average read count per SNP	10	100	10	10	**1, 2, …, 50**	10	0.5	0.5	0.5

**Figures**	Y-axis **Sensitivity, Precision**	**Number of SNPs per block**			**Average read count per SNP**			**Expected maternal reads fraction**		
	X-axis	S	M	S	S	M	S	S	M	S
Figure3A, left; Supplementary Figure3A	Overdispersion	10	100	10	10	10	10	0.5	0.9	0.5
Figure3B, left; Supplementary Figure3B, left	Center block length	10	**1, 2, …, 50**	10	10	10	10	0.5	0.9	0.5
Figure3B, center; Supplementary Figure3B, center	Average read count per SNP	10	100	10	10	**1, 2, …, 50**	10	0.5	0.9	0.5
Figure3B, right; Supplementary Figure3B, right	Expected maternal reads fraction	10	100	10	10	10	10	0.5	**0, 0.05, 0.10, …, 1**	0.5
	Y-axis **Specificity, Precision**									
	X-axis	S	S	S	S	S	S	S	S	S
Figure3A, right	Overdispersion	10	100	10	10	10	10	0.5	0.5	0.5
Figure3C, left	Center block length	10	**1, 2, …, 50**	10	10	10	10	0.5	0.5	0.5
Figure3C, right	Average read count per SNP	10	100	10	10	**1, 2, …, 50**	10	0.5	0.5	0.5
